# Significance of lymphovascular space invasion in epithelial ovarian cancer

**DOI:** 10.1002/cam4.31

**Published:** 2012-09-14

**Authors:** Koji Matsuo, Todd B Sheridan, Kiyoshi Yoshino, Takahito Miyake, Karina E Hew, Dwight D Im, Neil B Rosenshein, Seiji Mabuchi, Takayuki Enomoto, Tadashi Kimura, Anil K Sood, Lynda D Roman

**Affiliations:** 1 Division of Gynecologic Oncology, Department of Obstetrics and Gynecology, Los Angeles County Medical Center, University of Southern CaliforniaLos Angeles, CA; 2 Department of Pathology, Mercy Medical CenterBaltimore, MD; 3 Department of Obstetrics and Gynecology, Osaka University Faculty of MedicineSuita, Osaka, Japan; 4 Gynecologic Oncology Center, Mercy Medical CenterBaltimore, MD; 5 Department of Gynecologic Oncology, MD Anderson Cancer Center, University of TexasHouston, TX; 6 Cancer Biology, MD Anderson Cancer Center, University of TexasHouston, TX; 7 Center for RNA Interference and Non-Coding RNA, University of TexasHouston, TX; 8 Women's Cancer Program in Norris Comprehensive Cancer Center, Los Angeles County Medical Center, University of Southern CaliforniaLos Angeles, CA

**Keywords:** Lymph node metastasis, lymphovascular space invasion, ovarian cancer, survival

## Abstract

While the prognostic significance of lymphovascular space invasion (LVSI) is well established in endometrial and cervical cancer, its role in ovarian cancer is not fully understood. First, a training cohort was conducted to explore whether the presence and quantity of LVSI within the ovarian tumor correlated with nodal metastasis and survival (*n* = 127). Next, the results of the training cohort were applied to a different study population (validation cohort, *n* = 93). In both cohorts, histopathology slides of epithelial ovarian cancer cases that underwent primary cytoreductive surgery including pelvic and/or aortic lymphadenectomy were examined. In a post hoc analysis, the significance of LVSI was evaluated in apparent stage I cases (*n* = 53). In the training cohort, the majority of patients had advanced-stage disease (82.7%). LVSI was observed in 79.5% of cases, and nodal metastasis was the strongest variable associated with the presence of LVSI (odds ratio [OR]: 7.99, 95% confidence interval [CI]: 1.98–32.1, *P* = 0.003) in multivariate analysis. The presence of LVSI correlated with a worsened progression-free survival on multivariate analysis (hazard ratio [HR]: 2.06, 95% CI: 1.01–4.24, *P* = 0.048). The significance of the presence of LVSI was reproduced in the validation cohort (majority, early stage 61.3%). In apparent stage I cases, the presence of LVSI was associated with a high negative predictive value for nodal metastasis (100%, likelihood ratio, *P* = 0.034) and with worsened progression-free survival (HR: 5.16, 95% CI: 1.00–26.6, *P* = 0.028). The presence of LVSI is an independent predictive indicator of nodal metastasis and is associated with worse clinical outcome of patients with epithelial ovarian cancer.

## Introduction

Ovarian cancer remains a deadly disease and the most common cause of death among gynecologic malignancies. In 2012, over 22,300 women in the United States are estimated to be diagnosed with ovarian cancer, and over 15,500 will die of this disease [1]. The majority of ovarian cancer patients were present with advanced-stage disease, and cytoreductive surgery remains a mainstay in management [2]. Cytoreductive surgery for ovarian cancer includes total abdominal hysterectomy, bilateral salpingo-oophorectomy, pelvic and para-aortic lymphadenectomy, and omentectomy. Information obtained from the surgical specimen is useful for determining prognosis including histology, grade, and the extent of disease spread. However, given the imperfect predictive nature of these factors, additional markers are needed.

Lymphovascular space invasion (LVSI) is defined as the presence of tumor cells inside the capillary lumens of either the lymphatic or the microvascular drainage system within the primary tumor. The significance of LVSI has been extensively studied in other types of gynecologic malignancies such as endometrial [3, 4, 5, 6, 7], cervical [8, 9, 10, 11, 12, 13], and vulvar cancer [14, 15, 16]. In each, the presence of LVSI in the tumor is associated with increased risk of disease spread (especially nodal metastases), increased chance of disease recurrence, and decreased survival outcomes. In contrast, there has been little investigation on the impact of LVSI in epithelial ovarian cancer [17, 18], and the role of LVSI in the outcome of women with ovarian cancer remains unclear. The aim of this study is to evaluate the impact of the presence of LVSI within the ovarian malignancy on clinical variables and survival outcomes in women with ovarian cancer.

## Methods

### Training set cohort

After Institutional Review Board (IRB) approval was obtained in Mercy Medical Center in Baltimore, a previously established ovarian cancer database was utilized for this study [19]. Inclusion criteria included cases with epithelial ovarian cancer that underwent primary cytoreductive surgery including pelvic and/or aortic lymphadenectomy between January 1995 and January 2009. Cases with metastatic disease from sites other than ovarian primary, synchronous cancer types, and tumors of low malignant potential were excluded from the study. Variables abstracted from the medical records were patient demographics and survival outcomes after surgery.

The histopathology slides of the cases that met inclusion criteria were examined. The total number of slides stained with hematoxylin and eosin, the number of slides that contained ovarian tumor, and the number of slides with ovarian tumor that contained foci of LVSI were recorded for each case. The number of foci of LVSI was manually counted and the average number of foci of LVSI per slide was determined per case, and classified into “none” for no LVSI, and “low” (1–33 percentile), “moderate” (34–66 percentile), and “high” (≥67 percentile) among LVSI presenting tumors. For instance, if five slides contained LVSI within the ovarian tumor for a total of 10 foci, the average number of LVSI foci per slide was classified as 2 for that case. The gynecologic pathologist who evaluated the slides in this cohort was completely blinded to the clinical information. The total number of lymph nodes examined and the number of lymph nodes with tumor metastasis were also recorded. The presence and quantity of LVSI were then correlated with clinical variables, nodal metastasis, and survival outcome.

### Validation cohort

By utilizing the results of the training set cohort, an additional cohort was conducted and examined using a previously collected database for ovarian cancer in the participating institutions (Gynecologic Oncology Group in Osaka, Japan). IRB approval was obtained at each site. This study group was chosen to demonstrate whether the training set results were reproducible in a different population. Similar to the training set cohort, the histopathology slides of women with epithelial ovarian cancer who underwent primary cytoreductive surgery including pelvic and/or aortic lymphadenectomy were examined by different gynecologic pathologists who were blinded to the results of the training set cohort and to clinical information. Pulled slides were examined manually and the quantity of LVSI was scored as none, low, moderate, and high as defined by the training set cohort.

### Definition

Detailed description for the definition of LVSI is shown in Supplemental Methods. Among serous histology, grade 2 and 3 tumors were grouped as high-grade serous carcinoma analyzed as an independent group, whereas grade 1 tumors were grouped as low-grade serous carcinoma based on the recent accumulating data [20, 21]. In this two-tier grading system, it adequately correlates to conventional FIGO (the International Federation of Gynecology and Obstetrics) grading system and further provides valuable clinical outcome for ovarian cancer patients when compared with FIGO grading system demonstrating high-grade serous carcinoma as the distinct ovarian cancer subtype [20]. The significance of the presence of LVSI within the ovarian tumor was evaluated in apparent stage I disease across the two cohorts defined as ovarian tumor grossly confined to the ovary. The date of progression was determined by clinical examination, imaging studies, and/or CA-125 levels. Progression-free survival was defined as the time interval from the date of primary cytoreductive surgery to the date of documented first recurrence or progression of disease. If there was no recurrence, progression-free survival was determined as the date of last follow-up. Overall survival was defined as the interval between the primary cytoreductive surgery and the date of death or last follow-up.

### Statistical analysis

Continuous variables were assessed for normality (Kolmogorov–Smirnov test) and expressed as appropriate (mean with SD or median with range). Student's *t*-test or Mann–Whitney *U-*test was performed for continuous variable as appropriate. Categorical variables were evaluated with Fisher's exact test or chi-square test as appropriate, expressed with odds ratio (OR) and 95% confidence interval (CI). Risk factor of LVSI was evaluated with logistic regression test, and multivariate logistic regression test was further performed among significant variables in univariate analysis. Receiver–operator characteristic (ROC) curve analysis was performed to identify the risk factors for nodal metastasis expressed with area under the curve (AUC), and the cutoff analysis was performed to maximize the risk of nodal metastasis. Sensitivity, specificity, positive and negative predictive values, and accuracy of nodal metastasis were determined with the results of LVSI status. For survival data analysis, to determine the significance of variables for the survival outcomes for progression-free survival and overall survival, univariate (log-rank) and multivariate (Cox proportional hazard regression test) analyses were performed as appropriate. Survival curves were constructed with the Kaplan–Meier method. *P*-values of less than 0.05 were considered statistically significant (all, two-tailed). The Statistical Package for Social Science software (SPSS, version 12.0, IL) was used for all analyses.

## Results

**Figure 1:**
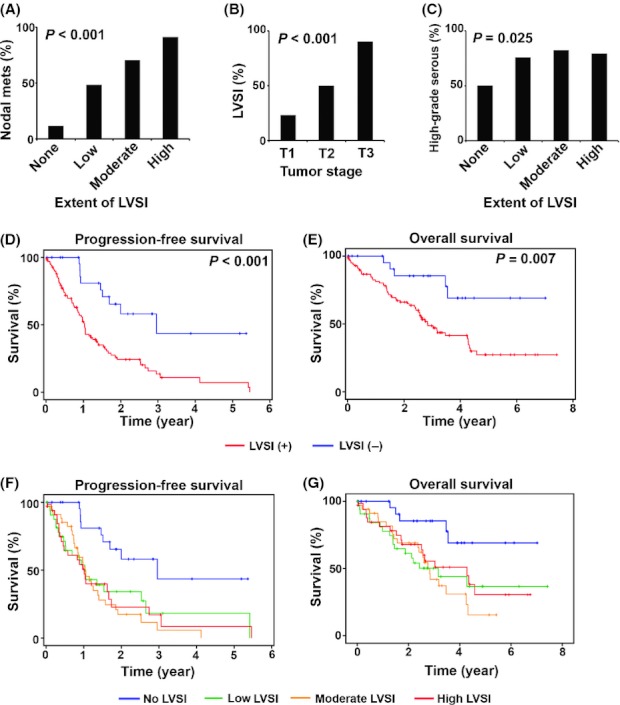
Lymphovascular space invasion and ovarian cancer in training set cohort. (A) Risk of lymph node metastasis based on the extent of LVSI is shown. (B) Correlation between tumor stage and LVSI. (C) Proportion of high-grade serous carcinoma is shown based on the extent of LVSI. (D) and (E) Survival curves based on LVSI status are shown. (F) and (G) Survival curves based on the extent of LVSI are shown. No LVSI, tumor expresses no LVSI, and low LVSI (1–33 percentile), moderate LVSI (34–66 percentile), and high (≥67 percentile) among LVSI presenting tumors in training set cohort. LVSI, lymphovascular space invasion; nodal mets, nodal metastasis.

The clinical characteristics of patients comprising the training set cohort are provided in Table 1. Mean age was 61 (±10.4), and the majority were Caucasian (89.0%), had advanced-stage disease (82.7%), and had high-grade serous tumors (73.2%). LVSI was noted in 101 (79.5%, 95% CI: 72.5–86.5) among 127 cases with the median number of LVSI foci per slide being 2 (range 0–53 foci, Kolmogorov–Smirnov's *P* < 0.001). Median number of slides containing LVSI was 5 (range 1–28) per case. In univariate analysis, nodal metastasis was associated with the presence of any LVSI (95.9% vs. 56.7%, *P* < 0.001), tumor stage (proportion of LVSI presenting tumor, T1 vs. T2 vs. T3: 23.1% vs. 50% vs. 90.2%, *P* < 0.001), and high-grade serous carcinoma (86.0% vs. 61.8%, *P* = 0.004) (Table 2, Fig. 1A–C). The magnitude of the significance of the presence of LVSI for nodal metastasis was similar between pelvic and aortic nodes (OR, 22.6 vs. 22.1). In multivariate analysis, tumor stage and nodal metastasis remained significantly associated with the presence of LVSI, and nodal metastasis was the strongest among significant variables (OR: 7.99, 95% CI: 1.98–32.1, *P* = 0.003, Table 2).

**Table 1:** Patient demographics in the two cohorts

	Training set cohort	Validation cohort	*P*
Cases	*n* = 127	*n* = 93	
Age	61 (±10.4)	52 (±8.8)	<0.001
Race			
White	113 (89.0%)	0	<0.001
Black	11 (8.7%)	0	
Asian	2 (1.6%)	93 (100%)	
Hispanic	1 (0.8%)	0	
FIGO stage			
I	11 (8.7%)	37 (39.8%)	<0.001
II	11 (8.7%)	20 (21.5%)	
III	90 (70.9%)	31 (33.3%)	
IV	15 (11.8%)	5 (5.4%)	
FIGO grade			
1	1 (0.8%)	24 (27.0%)	<0.001
2	20 (15.7%)	40 (44.9%)	
3	105 (82.7%)	25 (28.1%)	
Histology type			
Serous	94 (74.0%)	34 (36.6%)	<0.001
Endometrioid	7 (5.5%)	23 (24.7%)	
Clear cell	11 (8.7%)	24 (25.8%)	
Mucinous	2 (1.6%)	7 (7.5%)	
Others	13 (10.2%)	5 (5.4%)	
Two-tier grading system^1^			
High-grade serous	93 (73.2%)	28 (31.5%)	<0.001
Low-grade serous	1 (0.8%)	5 (5.6%)	
Other	33 (26.0%)	56 (62.9%)	
Nodal metastasis			
Pelvic lymph nodes^2^	66 (53.2%)		<0.001
Para-aortic lymph nodes^2^	31 (48.4%)		
Any lymph nodes	74 (58.3%)	17 (18.3%)	
Total slides examined^3^	41 (13–94)	18 (2–42)	<0.001
Slides for ovarian tumor^3^	9 (2–29)	7 (1–20)	0.001
Slides presenting LVSI^3^	5 (1–28)	2 (1-10)	<0.001
LVSI presenting tumor	101 (79.5%)	48 (51.6%)	<0.001

Mean (±SD), median (range), or number (%) is shown. FIGO, the International Federation of Gynecology and Obstetrics; LVSI, lymphovascular space invasion.

1 Grade 2 and 3 tumors are grouped as high-grade serous carcinoma, whereas grade 1 tumors are grouped as low-grade serous carcinoma among serous carcinoma.

2 In training set cohort, pelvic and para-aortic lymph nodes were evaluated in 124 and 64 cases, respectively.

3 Number of slides per each case. Grade: 4 and 1 cases missed in training and validation cohort, respectively.

**Table 2:** Variables associated with lymphovascular space invasion in ovarian cancer

		Univariate analysis		Multivariate analysis	
			
	Case number	OR (95% CI)	*P*	OR (95% CI)	*P*
Training set cohort					
Tumor stage (per stage)	13 vs. 12 vs. 103	6.54 (3.13–13.7)	<0.001	3.90 (1.46–10.4)	0.007
Any nodal metastasis (yes vs. no)	74 vs. 53	18.1 (5.06–65.0)	<0.001	7.99 (1.98–32.1)	0.003
Pelvic lymph nodes (yes vs. no)	66 vs. 58	22.6 (5.03–101)	<0.001		
Para-aortic lymph nodes (yes vs. no)	31 vs. 33	22.1 (2.68–182)	0.004		
High-grade serous carcinoma (yes vs. no)	93 vs. 34	3.81 (1.54–9.43)	0.004		0.78
Validation cohort					
Tumor stage (per stage)	40 vs. 22 vs. 31	2.45 (1.46–4.11)	0.001		0.16
Any nodal metastasis (yes vs. no)	17 vs. 76	9.77 (2.09–45.7)	0.004	5.74 (1.13–29.2)	0.035
High-grade serous carcinoma (yes vs. no)	28 vs. 61	3.37 (1.28–8.83)	0.014		0.16

Logistic regression test for presence of tumoral LVSI (yes vs. no). Examined all the collected variables and only significant variables are listed. OR, odds ratio; 95% CI, 95% confidence interval; LVSI, lymphovascular space invasion.

Next, the correlation between the quantity of LVSI and the risk of nodal metastasis was examined. Among 127 cases, 124 (97.6%) were available for pelvic lymph node evaluation and 64 (50.4%) cases for aortic lymph node evaluation. Median numbers of lymph nodes sampled from the pelvic and aortic areas were 4 (range 1–20) and 2.5 (range 1–11), respectively. In a predictive model with ROC analysis, the extent of LVSI significantly predicted lymph node metastasis (pelvic nodal metastasis, AUC 0.95, 95% CI: 0.91–0.99, *P* < 0.001; and aortic lymph nodal metastasis, AUC 0.93, 95% CI: 0.86–0.99, *P* < 0.001). To evaluate the significance of quantification of LVSI, a cutoff analysis was performed that showed that the presence of any LVSI (≥1 focus) was the strongest predictor of lymph nodal metastasis (OR for nodal metastasis per cutoff for LVSI foci per slide, 18.1 for 1 focus, 8.91 for 2 foci, and 11.9 for 3 foci, respectively). Among 101 cases containing LVSI, the quantity of LVSI was significantly correlated with the risk of nodal metastasis (*P* < 0.001). Based on the analysis, cases were classified into the following categories: none (LVSI, 0 foci), low (maximum 1 focus per slide per case), moderate (maximum 2 foci per slide per case), and high (3 or more foci per slide per case), respectively. Of cases with no LVSI, the proportion with lymph nodal metastasis was 11.5%, as compared with 48.5% in low cases, 70.6% in moderate cases, and 91.2% in high cases, (*P* < 0.001).

After a median follow-up time of 10.7 months, 85 (66.9%) women developed recurrence or progression of disease. On univariate analysis, significant predictors of progression-free survival were high-grade serous carcinoma (5-year rate, 5.0% vs. 41.3%, *P* = 0.004), FIGO stage (I, II, III, and IV: 76.2%, 67.5%, 6.4%, and 0%, *P* < 0.001), and presence of LVSI (8.3% vs. 47.1%, hazard ratio [HR]: 3.36, 95% CI: 1.67–6.74, *P* < 0.001) (Table 3 and Fig. 1D). For overall survival, FIGO stage (5-year rate, I, II, III, and IV: 100%, 79.0%, 28.7%, and 0%, *P* = 0.013) and presence of LVSI (26.5% vs. 67.7%, HR: 3.29, 95% CI: 1.32–8.24, *P* = 0.007) were significant predictors on univariate analysis (Fig. 1E). On multivariate analysis controlling for FIGO stage and high-grade serous carcinoma, the presence of LVSI remained a statistically significant variable for progression-free survival (HR: 2.06, 95% CI: 1.01–4.24, *P* = 0.048, Table 3). After controlling for FIGO stage, the presence of LVSI showed a trend toward worse overall survival although it did not reach statistical significance (HR: 2.16, 95% CI: 0.85–5.45, *P* = 0.10). Further analyses were performed on women whose ovarian tumors contained LVSI to determine whether the quantity of LVSI (low, moderate, or high) added further prognostic information. While the presence of tumoral LVSI was significantly associated with survival outcome (Fig. 1D and E), the quantity of LVSI present did not further impact progression-free survival (5-year rate, low, moderate, and high: 20.3%, 8.9%, and 0%, respectively, *P* = 0.84, Fig. 1F) or overall survival (*P* = 0.70, Fig. 1G).

**Table 3:** Lymphovascular space invasion and survival of women with ovarian cancer

		Univariate analysis		Multivariate analysis	
			
	Case number	HR (95% CI)	*P*	HR (95% CI)	*P*
Training set cohort					
Progression-free survival					
High-grade serous carcinoma (yes vs. no)	93 vs. 34	2.20 (1.27–3.83)	0.004		0.19
FIGO stage (per stage)	11 vs. 11 vs. 90 vs. 15	2.30 (1.60–3.30)	<0.001	1.95 (1.26–3.01)	0.003
LVSI (yes vs. no)	101 vs. 26	3.36 (1.67–6.74)	<0.001	2.06 (1.01–4.24)	0.048
Overall survival					
FIGO stage (per stage)	11 vs. 11 vs. 90 vs. 15	2.29 (1.45–3.60)	0.013	2.17 (1.31–3.60)	0.003
LVSI (yes vs. no)	101 vs. 26	3.29 (1.32–8.24)	0.007		0.1
Validation cohort					
Progression-free survival					
High-grade serous carcinoma (yes vs. no)	28 vs. 61	3.77 (1.90–7.46)	<0.001		0.26
FIGO stage (per stage)	37 vs. 20 vs. 31 vs. 5	3.05 (2.09–4.44)	<0.001	2.57 (1.59–4.00)	<0.001
LVSI (yes vs. no)	48 vs. 45	3.65 (1.72–7.78)	0.003	1.99 (0.90–4.20)	0.09
Overall survival					
High-grade serous carcinoma (yes vs. no)	28 vs. 61	2.84 (1.27–6.34)	0.008		0.75
FIGO stage (per stage)	37 vs. 20 vs. 31 vs. 5	2.54 (1.64–3.92)	<0.001	2.60 (1.48–4.57)	0.001
LVSI (yes vs. no)	48 vs. 45	3.35 (1.34–8.40)	0.006		0.18

Cox proportional hazard regression test. Examined all the collected variables and only significant variables are listed. HR, hazard ratio; 95% CI, 95% confidence interval; FIGO, the International Federation of Gynecology and Obstetrics; LVSI, lymphovascular space invasion.

### Validation cohort

**Figure 2:**
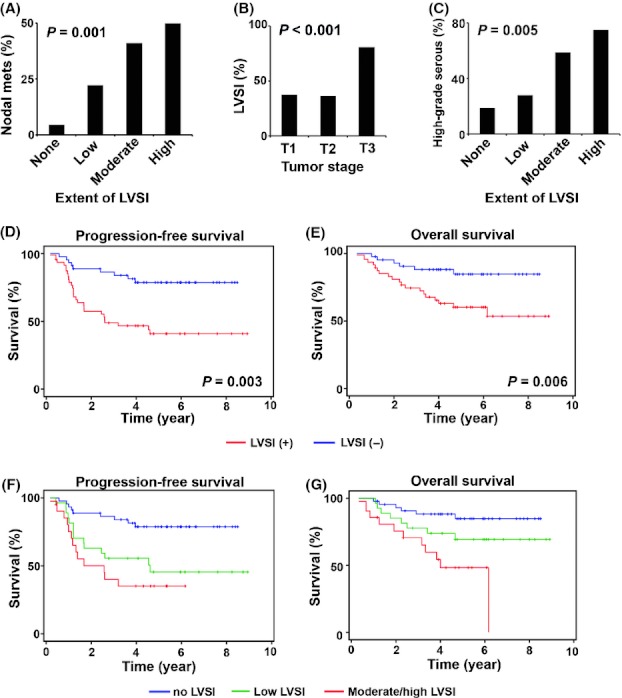
Significance of lymphovascular space invasion in validation cohort. (A) Risk of lymph node metastasis based on the extent of LVSI is shown. (B) Correlation between tumor stage and LVSI. (C) Proportion of high-grade serous carcinoma is shown based on the extent of LVSI. (D) and (E) Survival curves based on LVSI status are shown. (F) and (G) Survival curves based on the extent of LVSI are shown. No LVSI, tumor expresses no LVSI, and low LVSI (1 focus), moderate LVSI (2 foci), and high (≥3 foci) among LVSI presenting tumors in validation cohort. Cases with moderate and high LVSI were grouped due to small number in high (*n* = 4). LVSI, lymphovascular space invasion; nodal mets, nodal metastasis.

To determine whether our findings would be consistent in an independent cohort, we utilized a validation set of 93 cases from an entirely different study population. Patients in the validation cohort were younger, more likely to be of Japanese heritage, more likely to have early-stage disease, and less likely to have high-grade serous tumors (all, *P* < 0.001, Table 1). The presence of LVSI was noted in 48 (51.6%, 95% CI: 41.5–61.8) cases (low, moderate, and high LVSI: 56.3%, 35.4%, and 8.3%, respectively) and was less common than the frequency noted in the training set cohort (*P* < 0.001). In univariate analysis, the presence of LVSI was significantly correlated with nodal metastasis (AUC 0.77, 95% CI: 0.65–0.89, *P* < 0.001, Fig. 2A), tumor stage (T1, T2, and T3: 37.5%, 36.4%, 80.6%, *P* = 0.001, Fig. 2B), and high-grade serous carcinoma (71.4% vs. 42.6%, *P* = 0.013, Fig. 2C). Low-grade serous carcinoma showed significantly lower frequency of LVSI when compared with high-grade serous carcinoma (high-grade serous vs. low-grade serous vs. nonserous histology: 71.4% vs. 40% vs. 42.9%, *P* = 0.041). In multivariate analysis, the presence of LVSI remained a statistically significant predictive factor for nodal metastasis (OR: 5.74, 95% CI: 1.13–29.2, *P* = 0.035) after controlling for tumor stage and high-grade serous carcinoma (Table 2).

On univariate analysis, the presence of LVSI was associated with a worsened progression-free survival (5-year rate, 40.8% vs. 78.6%, HR: 3.65, 95% CI: 1.72–7.78, *P* = 0.0003) and overall survival (5-year rate, 59.6% vs. 85.0%, HR: 2.54, 95% CI: 1.64–3.92, *P* = 0.006) (Table 3, Fig. 2D and E). Similar to the training set cohort, the quantity of LVSI among ovarian tumors containing LVSI did not add further prognostic information: 5-year progression-free survival rate in low versus moderate/high, 45.7% versus 35.0%, respectively (*P* = 0.30, Fig. 2F); and 5-year overall survival rate, 68.9% versus 46.6%, respectively (*P* = 0.071, Fig. 2G). On multivariate analysis, the presence of LVSI remained as a marginally significant predictive factor associated with a worsened progression-free survival (HR: 1.99, 95% CI: 0.90–4.20, *P* = 0.09) after controlling for high-grade serous carcinoma (*P* = 0.26) and stage (*P* < 0.001). The presence of LVSI was not associated with overall survival in this study population (HR: 1.87, 95% CI: 0.74–4.85, *P* = 0.18) after controlling for high-grade serous carcinoma and stage.

**Figure 3:**
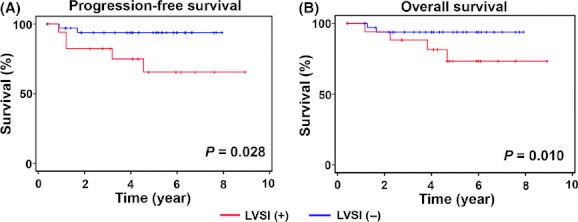
Significance of lymphovascular space invasion in apparent stage I ovarian cancer. (A) and (B) Survival curves for apparent stage I ovarian cancer based on tumor LVSI status. LVSI, lymphovascular space invasion.

A post hoc analysis of women with apparent stage I ovarian cancer across the two cohorts was performed to assess the potential impact of the presence of LVSI on nodal metastases and survival. There were 53 women with apparent stage I disease, of whom 18 (34.0%, 95% CI: 21.2–46.7) had tumors containing LVSI. The sensitivity, specificity, positive predictive value, negative predictive value, and accuracy of the presence of LVSI as a predictor of nodal metastases were 100%, 66.3%, 11.1%, 100%, and 68.5%, respectively (likelihood ratio, *P* = 0.034). The presence of LVSI was statistically significantly associated with decreased progression-free survival (5-year rate, 66.5% vs. 93.9%, HR: 5.16, 95% CI: 1.00–26.6, *P* = 0.028, Fig. 3A), but not overall survival (5-year rate, 72.6% vs. 94.0%, HR: 3.77, 95% CI: 0.69–20.6, *P* = 0.10, Fig. 3B). After controlling for high-grade serous carcinoma, the presence of LVSI showed a trend toward increased risk of poor progression-free survival (HR: 4.09, 95% CI: 0.75–22.4, *P* = 0.10).

## Discussion

The key findings of our study are that the presence of LVSI within the ovarian tumor is an independent predictive factor of both nodal metastasis and survival in women with ovarian cancer. If LVSI was present, the quantity of LVSI did not add further prognostic information, but it did impact the likelihood of nodal metastases. Our results add new information to the management of ovarian cancer exhibiting LVSI. Several key areas in the study deserve special mention.

In a view of systematic literature review using public searching engine PubMed and MEDLINE between 1955 and March 2012 with entry keywords of “ovarian cancer” and “lymphovascular space invasion,” there is little data evaluating the prognostic significance of the presence of LVSI in ovarian cancer [17, 18, 22, 23, 24]. These limited numbers of prior studies showed mixed results and were hampered by either small sample, lack of quantification of LVSI, or lack of a validation set. The summary of literature review is provided in Table S1. Collectively, the size, number, and quality of prior studies investigating the prognostic significance of LVSI in ovarian cancer were limited, and the impact of the presence of LVSI in ovarian cancer had not been clearly delineated.

Our study is the first to have a formal review of LVSI by a pathologist of all samples, presenting quantitative data, defining the role of LVSI, and validation in a separate independent cohort in epithelial ovarian cancer. The results demonstrated the strong link between the presence of LVSI and the likelihood of nodal metastases in women with ovarian cancer in two disparate study groups. In each, the quantity of LVSI present, as defined by maximum number of foci per slide, further correlated with likelihood of nodal metastases. When apparent stage I cases were combined in a post hoc analysis, the absence of LVSI within the ovarian tumor was an excellent predictor of negative nodal status. There are two possible clinical implications to these results. The first is that if surgical staging was incomplete in that lymphadenectomy was not performed, the lack of LVSI within the ovarian tumor gives reassurance that the nodes are uninvolved. Second, women with advanced-stage ovarian cancer whose tumors contain LVSI have a worsened progression-free survival and may benefit from consolidation therapy after completion of front-line chemotherapy. As vascular endothelial growth factor (VEGF) pathway is strongly associated with increased LVSI, targeting the VEGF axis with anti-VEGF inhibitor may be an attractive approach in ovarian tumor expressing LVSI [25, 26, 27].

A strength of our study is that the sample population is homogeneous in which only primary epithelial ovarian cancer cases who underwent primary surgery were included. Also, this is one of the largest studies evaluating the significance of the presence of LVSI in ovarian cancer. Furthermore, we demonstrated the durability of the impact of LVSI in two disparate cohorts. Potential weaknesses of the study are that it is retrospective in nature and thus confounding factors might have been missed, and that the sample size of women with apparent stage I disease is relatively small. In addition, only 50.4% cases of training set cohort have the information of aortic lymph nodes, and the number of lymph nodes sampled in our study was fewer than the reported literature. Therefore, nodal number may not be enough to evaluate the status of lymph node metastasis in apparent stage I ovarian cancer. Another limitation is that evaluation of the presence of LVSI in our study is based on hematoxylin and eosin staining but not on immunohistochemical analysis; however, it is the former that is widely used to determine the presence of LVSI. Prospective studies will be useful to help to confirm our findings, particularly in terms of the lack of nodal metastases seen in apparent stage I disease when LVSI is absent.

In conclusion, the presence of LVSI is an independent predictive indicator of nodal metastasis and is associated with a worse progression-free survival in ovarian cancer. Standardization of evaluation and scoring of LVSI would potentially yield important information that might help guide management. Further prospective investigation on the impact of the presence and quantity of LVSI in women with epithelial ovarian cancer is warranted.

## Supporting Information

**Table S1.** Results of systemic literature review for lymphovascular space invasion and ovarian cancer.
