# Supplementary material for: Significance of lymphovascular space invasion in epithelial ovarian cancer
Source: Cancer Med. 2012 Sep 14;1(2):156–64. doi: 10.1002/cam4.31 (PMC3544453; doi:10.1002/cam4.31)
Supplement: Supplementary file 1 [file cam40001-0156-SD1.doc]

**Significance of Lymphovascular Space Invasion in Epithelial Ovarian Cancer**

Koji Matsuo, MD^1,*^; Todd B. Sheridan, MD^2^; Kiyoshi Yoshino, MD, PhD^3^; Takahito Miyake, MD,PhD^3^; Karina E. Hew, MD^4^; Dwight D. Im, MD^4^; Neil B. Rosenshein, MD^4^; Seiji Mabuchi, MD, PhD^3^; Takayuki Enomoto, MD, PhD^3^; Tadashi Kimura, MD, PhD^3^; Anil K. Sood, MD^5,6,7^; Lynda D. Roman, MD^1,8^

1) Division of Gynecologic Oncology, Department of Obstetrics and Gynecology, and 8) Women’s Cancer Program in Norris Comprehensive Cancer Center, University of Southern California, Los Angeles County Medical Center, Los Angeles, CA, USA.

2) Department of Pathology, and 4) Gynecologic Oncology Center, Mercy Medical Center, Baltimore, MD, USA.

3) Department of Obstetrics and Gynecology, Osaka University Faculty of Medicine, Suita, Osaka, Japan.

5) Department of Gynecologic Oncology, 6) Cancer Biology, MD-Anderson Cancer Center, University of Texas, Houston, TX, USA.

7) Center for RNA Interference and non-Coding RNA, University of Texas, Houston, TX, USA.

*) All correspondence to:

Koji Matsuo, MD, Division of Gynecologic Oncology, Department of Obstetrics and Gynecology, University of Southern California, Los Angeles County Medical Center

2020 Zonal Avenue, IRD520, Los Angeles, CA 90031, Tel: +1-323-226-3416, Fax: +1-323-226-2743, Email: koji.matsuo@gmail.com

**Keywords:** ovarian cancer; lymphovascular space invasion; lymph node metastasis; survival.

**Running head:** LVSI and ovarian cancer

**Table S1. Results of systemic literature review for lymphovascular space invasion and ovarian cancer**

| **Authors** | **Year** | **No.** | **Type** | **Summary** |
| --- | --- | --- | --- | --- |
| O’Hanlan et al (17) | 1995 | 33 | Metastatic ovarian cancer to the gastrointestinal tract | Among cases with mesenteric nodal metastasis, 79% had LVSI noted within the tumor. Mesenteric nodal metastasis was marginally associated with LVSI (p=0.05). |
| Ariyoshi et al (20) | 2000 | 23 | Ovarian carcinosarcoma | LVSI within the primary tumor was seen in 8 (36.4%) out of 22 evaluable cases. LVSI was not associated with overall survival in the study (5-year overall survival rate, 16.7 versus 31.6%, p=0.46). |
| Fujimoto et al (21) | 2001 | 27 | Adult type granulosa cell tumors | LVSI was seen in 11 (40.7%) cases included minimal (n=6, 22.2%) and moderate/prominent (n=5, 18.5%), respectively. Disease-free survival of moderate/prominent LVSI were significantly shorter than that of none/minimal LVSI cases (p<0.0001). In multivariate analysis, moderate/prominent LVSI was associated with increased risk of recurrence (hazard ratio 8.2). |
| Nishimura et al (22) | 2005 | 36 | Endometrioid ovarian cancer | Compared 11 cases of endometrioid type ovarian cancer co-existing with endometrial cancer to 25 cases of endometrioid type endometrial cancer metastatic to ovary. Presence of LVSI within the primary tumor was not associated with survival (p=0.42) |
| Qian et al (18) | 2010 | 66 | Epithelial ovarian cancer | LVSI was seen in 36 (54.5%) of cases. LVSI was associated with advanced stage disease, high grade, and nodal metastasis (proportion of nodal metastasis and LVSI, none 23.3%, mild 22.2%, moderate 81.3%, and severe 81.8%, p<0.001). LVSI was an independent risk factor of disease-free survival (p<0.001) but not overall survival in multivariate analysis. |

Systematic literature review using public searching engine PubMed and MEDLINE between 1955 and March 2012 with entry keywords of “ovarian cancer” and “lymphovascular space invasion”. There are 5 results in the searching criteria.

**References**

17. O'Hanlan KA, Kargas S, Schreiber M, Burrs D, Mallipeddi P, Longacre T, Hendrickson M. Ovarian carcinoma metastases to gastrointestinal tract appear to spread like colon carcinoma: implications for surgical resection. Gynecol Oncol1995 Nov;59(2):200-6.

18. Qian X, Xi X, Jin Y. The grading of lymphovascular space invasion in epithelial ovarian carcinoma. Int J Gynecol Cancer2010 Jul;20(5):895-9.

20. Ariyoshi K, Kawauchi S, Kaku T, Nakano H, Tsuneyoshi M. Prognostic factors in ovarian carcinosarcoma: a clinicopathological and immunohistochemical analysis of 23 cases. Histopathology2000 Nov;37(5):427-36.

21. Fujimoto T, Sakuragi N, Okuyama K, Fujino T, Yamashita K, Yamashiro S, Shimizu M, Fujimoto S. Histopathological prognostic factors of adult granulosa cell tumors of the ovary. Acta Obstet Gynecol Scand2001 Nov;80(11):1069-74.

22. Nishimura N, Hachisuga T, Yokoyama M, Iwasaka T, Kawarabayashi T. Clinicopathologic analysis of the prognostic factors in women with coexistence of endometrioid adenocarcinoma in the endometrium and ovary. J Obstet Gynaecol Res2005 Apr;31(2):120-6.
