# Supplementary material for: Significance of lymphovascular space invasion in epithelial ovarian cancer
Source: Cancer Med. 2012 Sep 14;1(2):156–64. doi: 10.1002/cam4.31 (PMC3544453; doi:10.1002/cam4.31)
Supplement: Supplementary file 2 [file cam40001-0156-SD2.doc]

**Significance of Lymphovascular Space Invasion in Epithelial Ovarian Cancer**

Koji Matsuo, MD^1,*^; Todd B. Sheridan, MD^2^; Kiyoshi Yoshino, MD, PhD^3^; Takahito Miyake, MD,PhD^3^; Karina E. Hew, MD^4^; Dwight D. Im, MD^4^; Neil B. Rosenshein, MD^4^; Seiji Mabuchi, MD, PhD^3^; Takayuki Enomoto, MD, PhD^3^; Tadashi Kimura, MD, PhD^3^; Anil K. Sood, MD^5,6,7^; Lynda D. Roman, MD^1,8^

1) Division of Gynecologic Oncology, Department of Obstetrics and Gynecology, and 8) Women’s Cancer Program in Norris Comprehensive Cancer Center, University of Southern California, Los Angeles County Medical Center, Los Angeles, CA, USA.

2) Department of Pathology, and 4) Gynecologic Oncology Center, Mercy Medical Center, Baltimore, MD, USA.

3) Department of Obstetrics and Gynecology, Osaka University Faculty of Medicine, Suita, Osaka, Japan.

5) Department of Gynecologic Oncology, 6) Cancer Biology, MD-Anderson Cancer Center, University of Texas, Houston, TX, USA.

7) Center for RNA Interference and non-Coding RNA, University of Texas, Houston, TX, USA.

*) All correspondence to:

Koji Matsuo, MD, Division of Gynecologic Oncology, Department of Obstetrics and Gynecology, University of Southern California, Los Angeles County Medical Center

2020 Zonal Avenue, IRD520, Los Angeles, CA 90031, Tel: +1-323-226-3416, Fax: +1-323-226-2743, Email: koji.matsuo@gmail.com

**Keywords:** ovarian cancer; lymphovascular space invasion; lymph node metastasis; survival.

**Running head:** LVSI and ovarian cancer

**Supplemental Methods**

In training set cohort, we first set 3 methods to count lymphovascular space invasion (LVSI) in hematoxylin and eosin slides: *(i)* crude count, *(ii)* angiolymphatic vessels involved count, and *(iii)* equivocal count. In “crude count” method, it represents the number of foci of LVSI in the entire case (adding the number of foci from each slide together). Pathologists essentially counted every cluster of tumor cells within lymphovascular spaces except for the area for the equivocal as described below. For instance, if there are two foci occurred along the same plane and appeared to involve the same vessel, we considered it to involve the same vessel and scored that as a "2" in the LVSI crude number. On the contrary, in the method for “angiolymphatic vessels involved count”, this was counted as a “1”. Separately, for the areas that look like LVSI but unclear due to potential artifact or tumor cell contamination (whether the tissue appears torn in the area, if the slide has a lot of free tumor fragments along the edge of the tissue), it was defined as “equivocal count” and was not evaluated for the analysis. Then, receiver-operator-characteristic curve analysis was performed to predict any lymph node metastasis between the methods. The results indicated that both “crude count” method (AUC 0.89, p<0.001) and “angiolymphatic vessels involved count” method (AUC 0.89, p<0.001) were similar in predicting lymph node metastasis. Therefore, we chose “crude count” method for the further statistical analysis in the study. In post-hoc analysis, the AUC for “equivocal count” method was significantly smaller (AUC 0.62) than other two methods to predict lymph node metastasis, implying that these equivocal areas likely contain possible tumor contamination. In validation cohort, to evaluate whether or not the semi-quantification of LVSI is feasible across the evaluators, two pathologists scored the LVSI independently, and agreement of reading was examined with kappa statistics that showed statistical significance (p<0.001).
